# Using Intersectionality to Understand How Structural Domains Are Embedded in Life Narratives

**DOI:** 10.1111/jopy.12984

**Published:** 2024-11-02

**Authors:** Dulce Wilkinson Westberg, Moin Syed, Aerika Brittian Loyd, William Dunlop

**Affiliations:** ^1^ Department of Psychology University of California, Davis Davis California USA; ^2^ Department of Psychology University of Minnesota Minneapolis Minnesota USA; ^3^ Department of Psychology University of California, Riverside Riverside California USA

**Keywords:** ethnicity/race, gender, intersectional framework, life narratives, social class

## Abstract

**Objective:**

This study draws on life narrative data and an intersectional framework to explore features of narratives around structural domains, aiming to better understand the possible impacts of these domains on identity.

**Method:**

Through in‐depth semi‐structured interviews with 177 young adults from primarily minoritized groups (73% Asian American or Latine, 59% Women, Median Parent Income = $50,001 to $75,000), we gathered 885 life narratives. Young adults narrated a domain‐general, ethnic/racial, gender, social class, and intersectional experience. Features capturing the content (*Presence of Structural Domains*, *Connection to* and *Between Structural Domains*) and process (*Meaning Making*, *Affective Tone*) of narratives were explored.

**Results:**

Structural domains manifested uniquely within narratives such that ethnicity/race was discussed most frequently across narratives, whereas gender and social class were mentioned more in narratives about those domains. Additionally, *Meaning Making* was highest in self‐defining narratives and positively correlated with the number of structural domains present within and across narratives. *Affective Tone* was most positive in self‐defining narratives and most negative in social class narratives, which also contained the lowest *Connection to Structural Domain*.

**Conclusion:**

This study combines an intersectional framework and life narrative data to understand how structural domains manifest within young adults' experiences, revealing how those domains are interconnected and may impact identity.

## 1 Introduction

Personality psychology has been generally slow to respond to calls arguing that we must center structural contexts (e.g., racism) when examining psychological processes (Arshad and Chung 2022; Syed 2024; Westberg and Syed 2024). An *intersectional framework* highlights how structural domains including ethnicity/race, gender and social class are related (Crenshaw 1990), and can guide research on how identities and structures are interconnected. Accordingly, the current study draws from the intersectionality literature to explore how structural contexts are embedded in individuals' life narratives and may relate with processes of identity. Specifically, we explore the *content* of young adults' life narratives around structural domains, or *what* they talk about in their stories, in relation to the *processes* underlying their stories, or *how* they narrate their experiences (Galliher, McLean, and Syed 2017). In so doing, this study begins to provide foundational knowledge about how structural domains may guide life experiences and personality.

### 1.1 What Is an Intersectional Framework and How Have Psychologists Used It?

Intersectionality is a theoretical framework that has been used in psychology to draw attention to how systems of power and oppression imbue identity with meaning (Sabik 2016; Syed and Ajayi 2018). Black feminists and critical race theorists laid the basis for an intersectional framework (Collins 2019), and the term *intersectionality* emerged from Crenshaw's (1990) work examining how Black women were marginalized from a male dominated movement for Black rights and a feminist movement for white women.^1^ Work in this area highlights that structures of power including racism, sexism, and classism are fundamentally interconnected and impact experiences (Cole 2009; Settles and Buchanan 2014).

Unfortunately, research on intersectionality in personality psychology is limited and often misaligned with how the framework is used across the field (Westberg and Syed 2024). A recent review of US psychological research on intersectionality from 2005 to 2022 found that only four out of 555 papers on this topic appeared in personality‐focused psychological journals (Moffitt et al. 2023).^2^ Notably, these four papers relied only on quantitative data, even though 82% of the studies reviewed were qualitative. Relying exclusively on quantitative data is limited, as it does not allow researchers to explore how contexts are interconnected in experiences (Rogers, Niwa, et al. 2021). Personality psychologists' overreliance on statistical interactions among identity categories further compounds this limitation. Statistical interactions do not always imply meaningful interconnections among identities, nor do they account for structural power dynamics (Buchanan and Wiklund 2021). Beyond interaction effects, there is unique value in *listening* for relations between individuals and structures in people's narratives about their own experiences, a focus that is central to the current study (see also Rogers, Moffitt, and Jones 2021).

Applied to psychology, intersectionality can foster the increased use of a social justice lens, which reveals structural inequities and amplifies the voices of minoritized group members, to unpack how domains intersect in individuals' experiences (Settles and Buchanan 2014; Syed and Ajayi 2018). Thus, rather than examining how structural domains intersect at the societal level, the current study uses a social justice lens to explore how young adults view these domains in their own lives (see Rogers and Syed 2021). Demonstrating the importance of this perspective, Juan, Syed, and Azmitia (2016) identified differences in qualitative reflections about ethnicity/race and gender, finding that women of color were more likely to draw on personal rather than non‐personal experiences relative to white women. These findings highlight the importance of and need for additional research that critically considers how structural domains manifest through dynamics of power, rather than solely as collections of multiple identities (Buchanan and Wiklund 2021; Moradi and Grzanka 2017).

### 1.2 Applying an Intersectional Framework to Life Narratives

Life narratives are a component of personality capturing the storied representation of one's past, present, and imagined future that helps one make sense of themselves over time and across contexts, including how they align with cultural systems (McAdams and Pals 2006). Consequently, life narratives are ideal candidates for exploring the possible impacts of structural domains on identity. Past studies find that life narratives vary based on structural domains including ethnicity/race (Turner et al. 2022; Westberg 2022), gender (McLean and Breen 2009), and social class (Radmacher and Azmitia 2013). For example, Turner et al. (2022) found that Black American adults were more likely than white American adults to narrate experiences of perseverance in a dangerous world, illustrating how narratives may differ due to structural oppression. Moreover, Radmacher and Azmitia (2013) found that upwardly mobile poor and working‐class young adults narrated distinct strategies for making sense of class experiences. Taken together, work in this area provides evidence that individual differences in life narratives are shaped by specific identity domains, which can, in turn, serve as proxies for understanding broader structural influences (Quintana 2007; Settles and Buchanan 2014). But little research has explored how narratives reflect the intersection of multiple structural domains, such as how ethnicity/race and gender manifest in social class experiences and vice versa.

In this study, we use an intersectional framework to explore life narrative data, aiming to provide a descriptive basis for understanding structural domains in young adult's lives (Adler 2018; Westberg and Syed 2024). Specifically, we explore the ways in which structural domains are embedded within life narratives in relation to how these narratives are processed. Using prompts developed in past research, we gathered a domain‐general self‐defining narrative (Singer and Blagov 2004) and narratives about ethnicity/race (Syed and Azmitia 2008, 2010), gender (McLean, Shucard, and Syed 2017), social class (Radmacher and Azmitia 2013), and their intersections (Lilgendahl 2015; Westberg 2023). After examining the life narrative data and literature on intersectionality, we decided to examine features of narratives including *Presence of Structural Domains*, *Connection to Structural Domain*, *Connection Between Structural Domains*, *Meaning Making*, and *Affective Tone*. We examined narratives among a diverse sample of young adults, who past work indicates are likely to be engaged in identity work and more attuned to connections among such domains (Azmitia et al. 2023). Below, we offer theoretical rationale for investigating these narrative features and how they contribute to understanding of intersectionality within personality science.

#### 1.2.1 Presence of Structural Domains

As intersectionality emphasizes how individuals occupy multiple social positions that intersect to shape identity (Cole 2009; Crenshaw 1990), examining how structural domains emerge within individuals' life narratives is consistent with an intersectional framework (Rogers and Syed 2021). For example, one might narrate experiences with ethnicity/race through discussing gender expectations considered salient for members of their ethnic/racial group (Westberg 2022). In so doing, the person uses the lens of gender to unpack their experiences with ethnicity/race, demonstrating the two are intertwined (Juan, Syed, and Azmitia 2016). Therefore, the *Presence of Structural Domains* including ethnicity/race, gender, and social class may be significant psychological dimensions along which narratives vary.

Based on past research, we suspected that structural domains themselves may manifest within and across narratives in ways that guide understandings of the self. Although past research has not used an intersectional framework to examine structural domains in life narratives, it has demonstrated the utility of examining variations in how identity domains manifest in narratives. For example, Mayukha et al. (2024) identified distinct features in the domain‐general life narratives of individuals who mentioned ethnicity/race without prompting, arguing that spontaneous mentions revealed the importance of this domain to identity. Moreover, McLean et al. (2016) assessed the presence of eight life domains across narratives about those domains, finding that some were more likely to be mentioned across narratives than others. For example, the life domain of family was most likely to be mentioned in narratives across the eight domains, whereas sex roles were contained to narratives about that domain. Although we speculated that there would be a high mention of each structural domain in the narrative asking about that domain (e.g., gender will be mentioned highly in the gender narrative), we were uncertain the extent to which there would be mentions of each domain across narratives about different structural domains (e.g., mentions of gender within the ethnic/racial narrative). The domain‐general, self‐defining narrative represents a particularly intriguing case, as these are considered broadly representative of the self (Singer and Blagov 2004). Unprompted mentions of ethnicity/race, gender, and social class in self‐defining narratives may indicate particular importance of these structural domains to one's identity (Mayukha et al. 2024). Through assessing the *Presence of Structural Domains* within young adults' life narratives, we may gain an improved sense of how they are interconnected in their lives.

#### 1.2.2 Connection Within and Between Structural Domains

Assessing the *Presence of Structural Domains* is useful and important. However, mentioning structural domains in life narratives does not indicate the extent of one's connection to these domains or their perceived interconnection. Connection is a hallmark of identity (Dunlop and Westberg 2022), particularly when it comes to ethnicity/race and gender (Syed and McLean 2022). Past research indicates that connections with ethnicity/race and gender are positively associated with psychosocial adjustment (Rivas‐Drake et al. 2014; Rogers, Scott, and Way 2015). Given the significance of connection, it is essential to examine *Connection to Structural Domains* and how connecting to one domain might involve multiple domains. Emerging research suggests that personal connections to structural domains can prompt recognition of broader inequities, highlighting the need to examine these connections in life narratives (Mathews et al. 2020). In the current study, we examined connection (relative to disconnection) in narratives about structural domains to improve understanding of how young adults form these connections. We also assessed *Connection Between Structural Domains* in narratives about intersections among ethnicity/race, gender, and social class to enhance understanding of how these domains are perceived to be interconnected.

Relative to ethnicity/race and gender, what it means to feel connected with one's social class is less clear. Researchers find that while social class represents an important aspect of identity for young adults, connection with social class can manifest as upper‐class guilt as well as working‐class anger and pride (Thomas and Azmitia 2014). We hoped that through exploring connection to social class in life narratives, this study could provide additional insight into what social class connection might look like and its potential implications for identity.

#### 1.2.3 Meaning Making and Affective Tone

Content features of narratives including the *Presence of Structural Domains* and *Connection to* and *Between Domains* are valuable for understanding how domains are interconnected within people's experiences. But these features alone do not shed light on how young adults process experiences around structural domains. Two foundational features of narratives that capture variations in how individuals narrate experiences involving multiple domains are *Meaning Making* and *Affective Tone*.

*Meaning Making* in life narratives involves expressing self‐insight from life experiences that guides how one thinks, feels, and behaves in the world (McLean et al. 2020). In contrast, *Affective Tone* captures the positive to negative emotional valence of a narrative. Past research indicates that the process of *Meaning Making* is particularly important in life narratives that incorporate multiple domains. For example, McLean et al. (2016) found *Meaning Making* was more common in life narratives wherein participants discussed two or more identity domains. Additionally, researchers found that *Affective Tone* was an important feature of narratives involving multiple domains, as negative feelings of conflict between domains can prompt identity work (McLean, Shucard, and Syed 2017; Schachter 2004).

Although *Meaning Making* and *Affective Tone* may be relevant for understanding identity across domains, it remains unclear how these processes function in narratives about ethnicity/race, gender, and social class, or how they relate to the integration of multiple domains in one's narrative. Examining these identity processes in narratives within and across structural domains, and in relation to the number of domains young adults mention, can shed light on how young people process structural experiences and the possible interplay of multiple identities (Galliher, McLean, and Syed 2017; Lilgendahl 2015).

### 1.3 The Current Study

There is a need within personality psychology to flesh out what relations between persons and structures look like and how structures are interconnected within people's experiences using a social justice lens (Arshad and Chung 2022; Westberg and Syed 2024). This need is exacerbated by limited understanding of how structural domains of ethnicity/race, gender, and social class are reflected in lived experiences and how those types of experiences are processed (Galliher, McLean, and Syed 2017). To address these gaps, we draw on an intersectional framework to explore features of young adults' narratives around a domain‐general experience and experiences of ethnicity/race, gender, social class, and their intersections. The exploratory aim of this study was to better understand how structural domains are embedded within life narratives and their relationship with identity processes. In pursuit of this overarching aim, we address two research questions:

Research Question 1: How do features of narratives about domain‐general, ethnic/racial, gender, social class, and intersectional experiences manifest within and across narratives?

Research Question 2: How does the content‐oriented feature of *Presence of Structural Domains* relate to identity processes of *Meaning Making* and *Affective Tone*?

## 2 Method

Data were drawn from a larger study at a large university in Southern California examining “Your experiences in relation to various forms of cultural identity and psychological functioning (e.g., well‐being).” Approval for this two‐part study was obtained from the ethical review board at the university where data were collected (protocol number: HS ‐ 21‐004), including approval to use cloud‐based services for data management. Participants obtained one research credit for participating in an online survey and one research credit for participating in a semi‐structured interview. The narratives gathered during the semi‐structured interview are the focus of the current study.

### 2.1 Participants

Data collection occurred over a 4‐month period, during which time we aimed to recruit as many participants as possible. Our efforts resulted in a sample size of 177 young adults (*M*_age_ = 20.30, *SD* = 2.94, range = 18–38). This sample is large relative to existing narrative research examining multiple structural domains, yielding 885 narratives total (5 narratives from 177 participants).

We coded participants' forced‐choice and open‐ended responses of their ethnic/racial identification to sort them into one of five groups, resulting in a total of 68 Asian American, 63 Latine, 23 Multiracial, 16 white, and 6 Black/African American participants.^3^ The sample consisted of 104 women, 71 men, and 1 non‐binary participant. One participant did not provide demographic information including ethnicity/race and gender.

Participants reported a median parental household income of 5.00 (*M* = 4.80, *SD* = 2.12; 1 = Below $15,000, 2 = $150,001 to $25,000, 3 = $250,001 to $35,000, 4 = 35,001 to $50,000, 5 = $50,001 to $75,000, 6 = $75,000 to $100,000, 7 = $100,000 to $150,000, and 8 = Above $150,000). Additionally, the median of mother and father education was 1.00 (*M* = 1.50, *SD* = 0.67) and 1.00 (*M* = 1.58, *SD* = 0.78), respectively (1 = High school graduate, general education diploma, or some college, 2 = College graduate, 3 = Postgraduate degree, e.g., Masters, PhD, MD).

### 2.2 Procedure

Participants provided informed consent and completed a 50‐min online survey and a 60‐min semi‐structured interview in exchange for research credit. The online survey contained self‐report measures of personality and culture not used in the current study as well as prompts for basic demographic information. Although this study focuses on the life narrative data gathered from semi‐structured interviews, we also conducted analyses using self‐report measures from the online survey. These additional analyses are not included in this manuscript and are planned for a subsequent publication. The interview occurred on the webcam‐based platform, Zoom, within 2 weeks of the online survey. The first author conducted 122 interviews with young adult participants, and 55 interviews were conducted by trained research assistants. The semi‐structured interview contained narrative prompts that captured a domain‐general self‐defining narrative as well as narratives of ethnicity/race, gender, social class, and their intersections (see Table S2 for prompts). All narrative prompts, except the intersectional narrative, were derived from past research and have been shown to be suitable for life narrative research (McLean, Shucard, and Syed 2017; Radmacher and Azmitia 2013; Singer and Blagov 2004; Syed and Azmitia 2008). We developed the intersectional narrative prompt in response to calls to ask participants directly about intersections among identity domains to “capture critical psychological space in which identity processing takes place” (Lilgendahl 2015, p. 490).

### 2.3 Positionality

The authors are university faculty identifying as two cisgender women and two cisgender men. Regarding ethnicity/race, authors identify as Mexican American, Mixed‐ethnic, Black/African American, and white. The authors each have experience conducting research related to ethnic/racial identity among young adult college students from a social justice lens and with applying an intersectional framework. Five undergraduate researchers (identifying as: four women, one non‐binary person, three Asian American, and two white) contributed to qualitative data collection and/or analysis. As the researchers involved in data collection and analysis were students at the university where data were collected and most identified as women of color, our identities often overlapped with participants in ways that may have shaped our interactions and qualitative insights (Holmes 2021). Specifically, shared identities between researchers and participants may have provided reassurance to the latter, helping to promote openness and rapport during interviews. We acknowledge that this common ground may have granted us deeper insights into the qualitative data but also biased our interpretations of certain responses.

### 2.4 Narrative Features

During the semi‐structured interview, participants narrated a time when they felt aware of their (1) ethnicity/race and (2) social class, (3) a salient moment with gender, (4) an experience representing who they are across these domains (intersectional awareness), and (5) a domain‐general self‐defining narrative (Lilgendahl 2015; McLean, Shucard, and Syed 2017; Radmacher and Azmitia 2013; Singer and Blagov 2004; Syed and Azmitia 2008).^4^ The order of the ethnic/racial and social class awareness as well as the gender salient prompts was counterbalanced but the self‐defining and intersectional awareness prompts were always presented first and last, respectively. Recordings of spoken narratives from the interview were uploaded to Otter.ai, a software that converts speech to text transcription, and proofread for accuracy by trained research assistants. Narratives were then inputted into five spreadsheets, one for each prompt type, and randomized.

We identified features of narratives related to intersectionality based on the narratives themselves and the existing literature. Our coding approach followed existing guidelines for working with qualitative data (see Adler et al. 2017; Syed and Nelson 2015) such that the first author read a random subset of 10% of the narrative data and noted features that were unique to narratives for each prompt and shared across prompts. These notes were used to develop a coding manual of narrative features that the authors agreed targeted intersectionality including *Presence of Structural Domains*, *Connection to Structural Domain*, and *Connection Between Structural Domains*. In advance of reading the narratives, the author team decided to code for *Meaning Making* and *Affective Tone*, based on past research indicating that these processes are relevant when examining narratives about multiple identity domains (e.g., McLean et al. 2016).

Consistent with established practices in the published literature (Syed 2015), the first author created initial coding schemes based on existing research quantifying similar features (McLean et al. 2020). These coding schemes were refined and finalized during coding with trained research assistants. The first author coded all narratives alongside two teams of two research assistants who coded 60% of narratives for the purpose of interrater reliability. Two coders quantified features within self‐defining and gender narratives and two coders quantified features within ethnic/racial, social class, and intersectional narratives. Coding batches were distributed weekly in randomized spreadsheets containing 30, 40, and 38 narratives, each. Coding progressed based on prompt type and was recorded separately for each coder. In line with existing guidelines (Adler et al. 2017; Syed and Nelson 2015), coding teams met weekly with the first author until completion to discuss discrepancies and were able to change or maintain discrepant codes following a group discussion. Meeting regularly throughout the coding process helped prevent natural drift in coder's understanding of the narrative features (Syed and Nelson 2015). There was acceptable average interrater reliability for narrative features including *Presence of Ethnicity/Race* (*κ* = 0.97), *Gender* (*κ* = 0.95), and *Social Class* (*κ* = 0.96), *Connection to Structural Domain* (ICC = 0.72), *Connection Between Structural Domains* (ICC = 0.77), *Meaning Making* (ICC = 0.73), and *Affective Tone* (ICC = 0.74) across prompts where features were coded. Consistent with the “master coder” approach (Syed and Nelson 2015, 379), the first author's codes were used in quantitative analyses.^5^ The average length of participants' narratives ranged from 351 to 414 words across prompts. Regarding the intersectional narrative, most participants (*n* = 115) incorporated the domains of ethnicity/race, gender, and social class. But 40 participants focused on two identity domains, 9 participants focused on one identity domain, and 13 participants requested to skip this prompt. Below, we describe each narrative feature in greater detail (see Table S3 for examples of each feature).

#### 2.4.1 Presence of Structural Domains

The presence of ethnicity/race, gender, and social class was coded in all narratives using a presence/absence scale where 0 = Absent and 1 = Present. Participants who were unable to narrate experiences with certain domains received a code of “0” even if the domain was named. Coding for the mention of ethnicity/race, gender, and social class across narratives about these structural domains illustrated variability in how interconnected participant's experiences were.

#### 2.4.2 Connection to Structural Domain

The degree to which the narrator described a connection to ethnicity/race, gender, and social class in the narrative about that domain was coded on a three‐point scale ranging from 1 = Disconnected, 2 = Neither Connected or Disconnected, and 3 = Connected. This narrative feature and its coding scheme take after the commonly coded narrative feature of communion (McLean et al. 2020). While communion captures broad feelings of connection or isolation with others and the world, *Connection to Structural Domain* is a more domain‐specific version that reflects connection or isolation with ethnicity/race, gender, and social class in narratives about those experiences. The scale we used to capture *Connection to Structural Domain* within ethnic/racial, gender, and social class narratives resembles past research quantifying communion (Adler et al. 2015). However, our scale was condensed to three points to better align with the level of variability present in young adults' narratives related to a single structural domain. Participants must have elaborated on feelings of disconnection or connection to the structural domain being discussed to receive a “1” or “3” for this feature, respectively. For example, one participant who expressed, “it was sad being told that it wasn't obvious I was Filipino. It made me feel less than or not as Filipino” received the code of “1” (disconnected). Thus, higher *Connection to Structural Domain* could involve feelings of belonging, integration, or solidarity with one's group, whereas lower levels of this feature could involve feelings of isolation, marginalization, or detachment from one's group. Accordingly, narratives with lower *Connection to Structural Domain* tended to contain more negative tone on average (*r* = 0.50, *p* < 0.01, 95% CI [0.38, 0.61]), though there was not a required tone for this feature to be coded as high or low in narratives.

#### 2.4.3 Connection Between Structural Domains

We also assessed the extent to which participants expressed connection relative to disconnection between each of the structural domains discussed in the intersectional narrative. Like the scale we used to quantify *Connection to Structural Domain* in the ethnic/racial, gender, and social class narratives, the scale we used to capture *Connection Between Structural Domains* is consistent with past research quantifying communion in narratives, more broadly (Adler et al. 2015). However, rather than the condensed three‐point scale used to capture connection to one structural domain, we used the full five‐point scale ranging from 1 = Highly Disconnected, 2 = Disconnected, 3 = Neither Connected or Disconnected, 4 = Connected, and 5 = Highly Connected. Although the three‐point scale was appropriate for capturing connection to one structural domain, the five‐point scale was used here to accommodate greater variability in participants' responses regarding multiple identity domains in the intersectional narrative. Narratives with higher disconnection did not integrate structural domains and focused on how domains were disconnected, whereas narratives with high connection highlighted meaningful interconnections among domains. Narratives with higher *Connection Between Structural Domains* tended to be more positive in tone (*r* = 0.23, *p* < 0.01, 95% CI [0.09, 0.37]), although this correlation was smaller in magnitude compared to the one observed for *Connection to Structural Domain*.

#### 2.4.4 Meaning Making

In line with past research (McLean et al. 2020), we coded all narratives for *Meaning Making* on a four‐point scale including: 0 = No explanation about meaning of event, 1 = Lesson learned from event, 2 = Some growth or change in the self but specifics of the change are unclear, and 3 = Evidence that the narrator gleaned specific insight from the event that applies to broader areas of their life. Higher scores were indicative of greater meaning making.

#### 2.4.5 Affective Tone

Consistent with past research (McLean et al. 2020), positive relative to negative emotional tone was coded in all narratives on a five‐point scale ranging from 1 = Very negative to 5 = Very positive, with 3 = Neutral. Narratives with higher negative relative to positive affective tone typically mentioned more negative events and focused on negative emotions.

### 2.5 Transparency and Openness

Researcher codes of narrative features, analysis code, and study materials are available here: https://osf.io/6zn4j/. Qualitative data are not made publicly available due to risk of reidentification. Analyses were conducted in R v.4.2.2 (R Core Team 2021) via RStudio v.2023.6.2.561, using the *psych* (Revelle 2024), *dplyr* (Wickham, Vaughan, and Girlich 2024), *tidyr* (Wickham, Vaughan, and Girlich 2024), *lme4* (Bates et al. 2015), and *ggplot2* (Wickham 2016) packages. This study's design and its analysis are exploratory and were not preregistered.

### 2.6 Analysis Plan

For each research question, we present narrative examples to illustrate how specific features manifested. The reasons for presenting qualitative data examples are two‐fold. First, these data provide rich, contextual insights into young adults' lived experiences, allowing us to show how narrative features manifested with consideration of structural inequities. Second, by grounding our results in young adults' own words and stories, we reinforce our quantitative findings and ensure that the voices of minoritized individuals are central to our research (Rogers and Syed 2021; Westberg and Syed 2024). Our approach exemplifies a commitment to a social justice lens through considering how structural factors influence personal narratives and makes the features themselves more tangible and applicable. Thus, this study addresses calls for research to critically examine how structural domains are embedded within life experiences and may inform personality (Arshad and Chung 2022). It is important to note that the narrative examples we present are not considered to be representative of all young adults, but they are useful insofar as they represent the site at which quantitative findings of this study are substantiated and help illustrate the experiences provided by young adults in our study.

To address research question 1, we assessed how *Presence of Structural Domains*, *Connection to Structural Domain*, *Connection Between Structural Domains*, *Meaning Making*, and *Affective Tone* varied based on prompt type and participants' self‐identified ethnicity/race, gender, and social class. We first report the frequency with which each structural domain is mentioned (i.e., *Presence of Structural Domains*) based on narrative prompt. Although the *Presence of Structural Domains* within narratives about that domain is likely to be high, we used binomial tests to assess whether the distribution of mentions across prompts significantly deviated from chance. Next, we used repeated measures ANOVAs to examine variations in average levels of *Connection to Structural Domain*, *Meaning Making*, and *Affective Tone* based on narrative prompt. We did not examine variations in *Connection Between Structural Domains* based on prompt, as this was only quantified in the intersectional narrative. Lastly, we used multiple regression to examine whether narrative features averaged across all prompts in which they were coded varied based on participants' self‐identified ethnicity/race, gender, and social class. Based on the sizes of each ethnic/racial group, we only examined statistical differences between Asian American and Latine participants. Additionally, we used a standardized (z scored) aggregate of parental income and education when examining variations based on social class to provide insight into the overall effect of objective social class (Kraus, Piff, and Keltner 2009).^6^ One participant who did not provide demographic information was excluded from these analyses and one participant who identified as non‐binary was excluded in analyses examining gender differences.

To address research question 2, we examined whether process‐oriented features of *Meaning Making* and *Affective Tone* correlated with the number of structural domains the narrator mentioned within and across their life narratives. As this involved examining correlations among narrative features, we employed partial correlations controlling for the length in words of participants' narratives. First, we examined partial correlations between *Meaning Making* and *Affective Tone* and the number of domains mentioned within each narrative (range = 0–3 domains). Next, we examined bivariate correlations between these features and the mean number of domains mentioned across all narratives (range = 0.5–1.75).

## 3 Results

Here, we present results on how each narrative feature manifested based on narrative prompt and self‐identified ethnicity/race, gender, and social class (research question 1). We also report whether *Meaning Making* and *Affective Tone* related with the *Presence of Structural Domains* (research question 2). Throughout, we provide narrative examples of how each feature emerged within life narratives. Means, standard deviations, and range of each narrative feature are reported in Table 1.

**TABLE 1:** Means and standard deviations of narrative features.

Narrative prompt	Ethnicity/race	Gender	Social class	Meaning making	Affective tone	Connection
Self‐defining						
*M*	0.23	0.09	0.13	2.29	3.25	NA
*SD*	NA	NA	NA	0.89	1.17	NA
Range	0–1	0–1	0–1	0–3	1–5	NA
Ethnicity/race						
*M*	0.99	0.08	0.08	1.93	2.90	2.38
*SD*	NA	NA	NA	1.01	1.20	0.84
Range	0–1	0–1	0–1	0–3	1–5	1–3
Gender						
*M*	0.21	0.98	0.06	1.90	2.97	2.38
*SD*	NA	NA	NA	1.08	0.99	0.74
Range	0–1	0–1	0–1	0–3	1–5	1–3
Social class						
*M*	0.11	0.02	NA	1.92	2.54	1.99
*SD*	NA	NA	NA	0.92	0.92	0.86
Range	0–1	0–1	NA	0–3	1–5	1–3
Intersectionality						
*M*	0.90	0.77	0.79	1.65	2.74	3.32
*SD*	NA	NA	NA	1.13	1.14	1.26
Range	0–1	0–1	0–1	0–3	1–5	1–5

*Note: N* = 170–177. “Connection” denotes *Connection to Group* in ethnicity/race, gender, and social class narratives and *Connection Between Domains* in the intersectionality narrative. Social class was mentioned within all social class narratives.

### 3.1 Research Question 1: Features Within and Across Narratives

#### 3.1.1 Presence of Structural Domains

**FIGURE 1:**
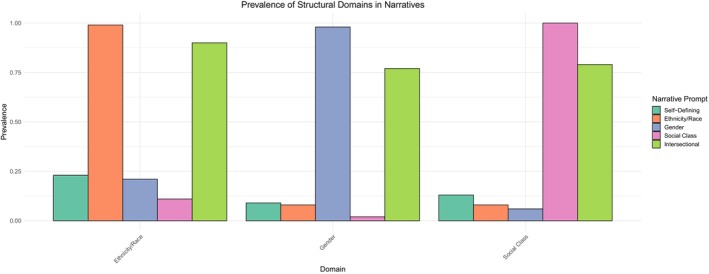
Prevalence of ethnicity/race, gender, and social class across the five narrative prompts.

To address research question 1, we examined the prevalence of each narrative feature within and across narrative prompts. We then examined whether narrative features varied based on participants' self‐identified ethnicity/race, gender, and social class. Figure 1 summarizes the frequencies of structural domains within and across narrative prompts. As expected, ethnicity/race and gender were mentioned in nearly every ethnic/racial and gender narrative, respectively, and social class was mentioned in all social class narratives. While there was not much variability in the mention of a structural domain in the prompt about that domain, there was variability in the mention of a domain in response to prompts about domain‐general and other domains. Specifically, participants mentioned ethnicity/race moderately within self‐defining (23%) and gender narratives (21%) and less in social class narratives (11%). In contrast, gender was mentioned less often across self‐defining (9%), ethnic/racial (8%), and social class narratives (2%). Social class was mentioned relatively infrequently in self‐defining narratives (13%) and rarely in ethnic/racial (8%) and gender narratives (6%). We examined the likelihood of these descriptive results by testing them via a binomial test in which we set the expected frequency of each to 10%.^7^ These tests indicated that ethnicity/race was more likely than chance to be mentioned in self‐defining (*p* < 0.001) and gender (*p* < 0.001) narratives. Gender was *less* likely to be mentioned by chance in the social class narratives (*p* < 0.001). All other tests were not significant. Based on the reported frequencies, ethnicity/race held a higher degree of relevance across narratives about various structural domains. For example, Grace, a Chinese American woman (objective social class = −0.94), shared in her self‐defining narrative,

I was raised by immigrant parents. They came here [in] the 1970s and started a restaurant. Growing up, I would hang around the restaurant all the time while [my parents] watched me. They would feed me, help with homework, all while taking care of the restaurant. That was one of the pivotal moments in my life where I realized my parents multitask everywhere. That created a sense of “I'm in debt to my parents.” The reason I work hard or stay motivated is because I feel a sense of debt to them. I've watched them live a hard life. That's one of my critical moments. I wake up every day, and that gives me motivation to do more. To go out there and create a better future for my parents because they were immigrants and coming here, they're pursuing the American dream, hoping that their child would be educated, well rounded, smart, like ideal. Honestly, I don't embody all of those. But I still feel the need to give my parents an easier life and fulfill that. Be a “good kid.” That made me who I am today.

Grace describes navigating ethnicity/race through experiences with immigration, including her parents' desire to fulfill the “American dream” and perceived expectations around what it means to be Asian American (Yip et al. 2021). Her narrative is layered with a deep understanding of her parents' struggles, which motivate her to pursue higher education and create a better future for her family (Schwartz et al. 2010). Through her narrative, Grace describes a sense of who she is and where she would like her life to go in relation to her ethnicity/race, showing how structural domains including ethnicity/race are significant aspects of young adults' experiences that might shape their personality including their goals and identity.

Gender and social class were more “contained” relative to ethnicity/race, with them being mentioned at a lower rate outside narratives about their own domain and the intersectional narrative. However, manifestations of ethnicity/race and gender in particular illustrated interlocking structures of oppression (Crenshaw 1990; Juan, Syed, and Azmitia 2016). For example, in her self‐defining narrative, Sofia, a Mexican American woman (objective social class = −0.46), shared how the intersection of ethnicity/race and gender shaped her experience with family, noting,

The experience that I feel defines the person I am today is that I grew up in a Hispanic household and the mentality is that women are weaker. There was this one time where my dad told me to do something, but I was like, oh, he could do it themselves. So, I told him that. He got mad and hit me. After that event, I got mad. I started hating how they thought. Now I'm really trying whenever I meet someone, if they have that mentality, that women are weaker or women need to do this and that I tend to get away, because I don't want to go through that. I feel like it's made me more independent. I can do so much more than this mentality that has been present throughout my whole life.

Sofia's father sought to uphold oppressive standards of what he felt a Hispanic woman ought to be, standards that are both sexist and racist. Through her self‐defining narrative, Sofia describes resisting power dynamics associated with her ethnicity/race and gender. This is consistent with recent research showing that challenging the status quo may be a significant aspect of identity, particularly for youth from minoritized groups (Rogers, Niwa, et al. 2021; Syed and McLean 2022).

#### 3.1.2 Connection to and Between Structural Domain(s)

**FIGURE 2:**
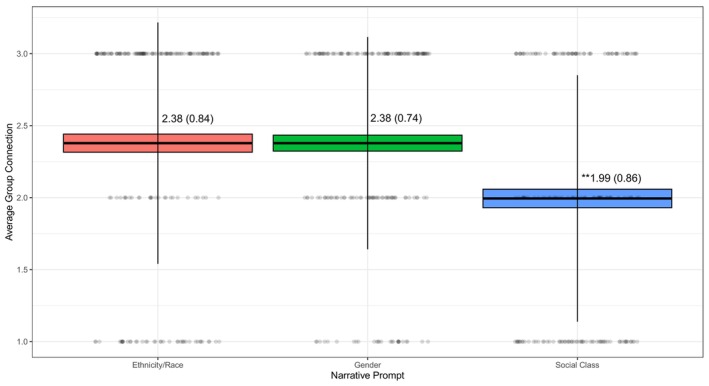
Variations in connection to structural domain by narrative prompt. *Note:* Range of connection to structural domain = 1–3. Values represent M (SD). **Significant difference at *p* < 0.01 derived using Tukey's HSD.

We examined *Connection to Structural Domain* within the ethnicity/race, gender, and social class narratives and *Connection Between Structural Domains* within the intersectional narrative. We tested statistical differences in mean‐levels of *Connection to Structural Domain* based on narrative prompt using repeated measures ANOVA. We found that there were differences in *Connection to Structural Domain* across narratives (*F*(2, 176) = 13.37, *η*_*p*_ = 0.05, *p* < 0.01) such that this feature was lower within social class narratives (*M* = 1.99, *SD* = 0.86) relative to ethnic/racial (*M* = 2.38, *SD* = 0.84) and gender narratives (*M* = 2.38, *SD* = 0.74; see Figure 2).

Young adults who evinced greater *Connection to Structural Domain* within narratives about those domains often discussed how they felt connected despite challenging power dynamics. For example, Luis, a Latino man (objective social class = −0.31), expressed connection to ethnicity/race through his gender in his ethnic/racial narrative, sharing,

This guy made fun of my mustache. It was pretty dark, and [he] would make fun of me. He's like, “Dude, you have facial hair. You're 13. That's weird.” So, I shaved. I got rid of my mustache. I wish I hadn't done it. That guy made fun of me because I had facial hair, and I shaved it. And it got worse, right? I got to the point where I'd shave every morning to have a clean face. I realized I didn't want to keep shaving. Hair is not a bad thing. It's natural. Some people have less. Some people have lighter hair, so you can't really see it. The way I am, I should keep it. That's when I realized I'm shaving my chest, arms, face, to look like somebody who I will never look like. I'll never be like. Who I don't need to look like and don't need to be like. Throughout high school trying to be somebody I'm not made me realize who I was. Accept it, be proud of it.

Through his narrative, Luis rejects white beauty standards and describes embracing his identity as a Latino man. His story shows that connection with one's ethnicity/race through gender can function as a source of strength and supports past research finding that ethnic/racial and gender identity are interconnected (Rogers, Scott, and Way 2015). Narratives like Luis's also provide additional evidence that mentioning structural domains can involve resistance of structural norms (i.e., white beauty standards) and bolster feelings of liberation and growth (Rogers and Syed 2021).

In contrast, social class narratives often involved recognizing socioeconomic differences between oneself and others (i.e., outgroup comparisons; Radmacher and Azmitia 2013). Participants tended to evince lower connection to social class when they focused on challenges arising from it, and higher connection when discussing personal growth. For example, Ava, a Multiracial woman who identified as Chinese Mexican American (objective social class = −0.94), expressed low connection to social class in her narrative about this domain, sharing,

There was one time I was very young, but we were going to a friend's birthday party. My parents felt the obligation to buy gifts for the birthday girl. I remember choosing out a Barbie doll for that girl and feeling really bad that my parents couldn't afford a new toy for myself. But we were buying this new toy for another girl, and it just felt really bad. I'm sorry. It made me realize that it doesn't matter how hard my parents' work. Money was always an issue. They were always working so hard, yet we can't afford things that we wanted. It made me see the world differently. That it's not fair. People are working less, but they are earning more money than my parents. I came to realize that education and opportunities in life are given to specific people.

Ava's narrative displays an increased awareness of economic inequality and societal inequities and demonstrates how forming connections to one's social class is relatively more complex (Radmacher and Azmitia 2013). In contrast, Monique, a Lebanese woman (objective social class = −0.46), expressed connection to social class in her narrative about this domain, sharing,

My dad came here at a very young age. He worked to make a living. So, I always try to remember where I came from. I grew up in a humble, modest home. I think people from the upper class sometimes can seem a bit degrading. Sometimes you feel a bit uncomfortable around these people because they have this arrogance, and they think that they're above you just because of their financial circumstances. I've had those experiences before.

From this, we see that Monique is aware of differences between her own social class and that of others. This awareness fosters a sense of connection with her social class. She goes on to note,

But the way I look at it now, I've definitely grown and become more aware of myself. I always remember where I came from, and how much my parents sacrifice and struggle to get a job. They worked hard to raise money and provide for us. The way I see social class is that I always try to be as humble as possible, because everyone has a story.

By the conclusion of her narrative, we see that Monique's unique family history shapes her view of social class and her desire to be humble and respectful of other people's circumstances. Her narrative reveals that personal growth may be important for forming positive connections with one's social class.

The narrative feature *Connection Between Structural Domains* was only captured in the intersectional narrative and was moderate overall (*M* = 3.32, *SD* = 1.26, range = 1–5). Because this feature was only represented in one narrative, we did not examine variations across prompts, but the distribution indicates wide variability in how individuals perceive connections among these domains. Based on the narrative data, we see that *Connection Between Structural Domains* sometimes involved navigating interlocking structures of racism, sexism, and classism (Crenshaw 1990; Juan, Syed, and Azmitia 2016). For example, Aaliyah, a Black woman participant with high *Connection Between Structural Domains* in her intersectional narrative, (objective social class = 0.12) reported,

Being a person of color at a private school, was looked at confusingly, “How can you afford this? You're Black. You should go down to the public school where the rest of you are.” Well, dang. In my mind, I'd seen successful Black people in my family. I never thought Black people are inherently poor. There's plenty of successful Black people. That was the first time where race and economic status felt intertwined to me. Another one was being Black and male. I didn't have any experience with Black women until high school. For a long time, my experience being Black was the male experience. It took me a long time to realize the Black female experience is completely different because they're fighting on two fronts. Being oppressed on two fronts. Being a woman and being Black. When I saw race, it was always a Black man. Look at the civil rights movement, and all these things we learned in history. It's the Martin Luther King's, it's the Malcolm X's, the John Lewis's. I was in college when I started to hear about female activists and leaders. It shouldn't have taken until college to realize. But when I did, it was eye opening.

Women participants, especially those of color who are well‐represented in this study (84% of women identified as Asian, Black, or Latinx), may have a higher threshold for connection of ethnicity/race, gender, and social class based on interconnected experiences of racism, sexism, and classism (Juan, Syed, and Azmitia 2016). This pattern was evident in the narratives of multiple women of color participants and reveals that high *Connection Between Domains* may involve recognizing interlocking structures of oppression.

#### 3.1.3 Meaning Making and Affective Tone

**FIGURE 3:**
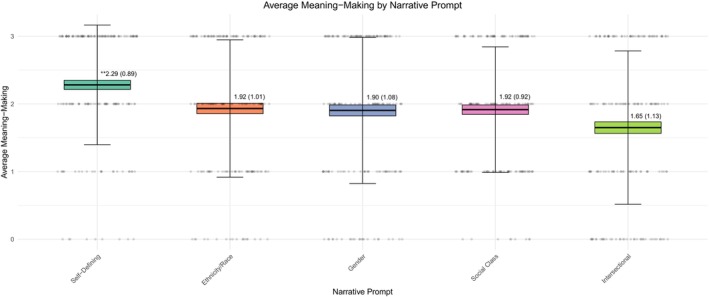
Variations in meaning making by narrative prompt. Range of meaning making = 1–5. Values represent *M* (*SD*). **Significant difference at *p* < 0.01 derived using Tukey's HSD.

Features including the *Presence of Structural Domains* and *Connection to Structural Domain* reveal the descriptive landscape of young adult's narratives. However, features including *Meaning Making* and *Affective Tone* clarify how young adults process these experiences and incorporate them into their identity. Results from a repeated measures ANOVA (*F*(4, 172) = 8.81, *η*_*p*_ = 0.04, *p* < 0.01) indicated that *Meaning Making* was significantly higher in the self‐defining narrative (*M* = 2.29, *SD* = 0.89) relative to all other narrative prompts (see Figure 3). On average, participants reported moderate levels of *Meaning Making* in the ethnic/racial, gender, and social class narratives.

**FIGURE 4:**
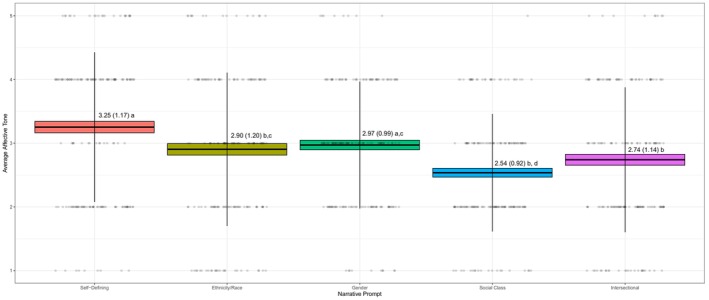
Variations in affective tone by narrative prompt. Range affective tone = 1–5. Values represent *M* (*SD*). Subscripts that differ from one another within the same row indicate significant difference at *p* < 0.01 derived using Tukey's HSD.

*Affective Tone* also varied based on narrative prompt (*F*(4, 172) = 10.60, *η*_*p*_ = 0.04, *p* < 0.01). Specifically, self‐defining narratives were more positive (*M* = 3.25, *SD* = 1.17) relative to ethnic/racial (*M* = 2.90, *SD* = 1.20), social class (*M* = 2.54, *SD* = 0.92), and intersectional narratives (*M* = 2.74, *SD* = 1.14). In addition, ethnic/racial and gender narratives were more positive (*M* = 2.97, *SD* = 0.99) relative to social class narratives, making social class narratives the most negative overall (see Figure 4).

#### 3.1.4 Variations in Features Based on Self‐Identified Ethnicity/Race, Gender, and Social Class

We used multiple regression to examine mean differences in features based on participants' self‐identified ethnicity/race, gender, and social class. Due to group sizes, we only examined differences between Asian American and Latine participants. We found no evidence for variations in features averaged across prompts including *Presence of Structural Domains*, *Connection to Structural Domains*, and *Connection Between Structural Domains* based on self‐identified ethnicity/race, gender, or objective social class (Tables S4–S8). But we found a difference in *Affective Tone* based on gender such that men participants provided more positive narratives on average during their interviews relative to women participants (*β* = −0.26, *p* = 0.01).

### 3.2 Research Question 2: Linking Content and Process Features

To address research question 2, we examined whether *Presence of Structural Domains* within and across life narratives correlated with *Meaning Making* and *Affective Tone*, while controlling for the length in words of participants' narratives.^8^ We found that *Meaning Making* correlated positively with the number of structural domains present within narratives about ethnicity/race, gender, social class, and their intersections, but not in the self‐defining narrative.

**FIGURE 5:**
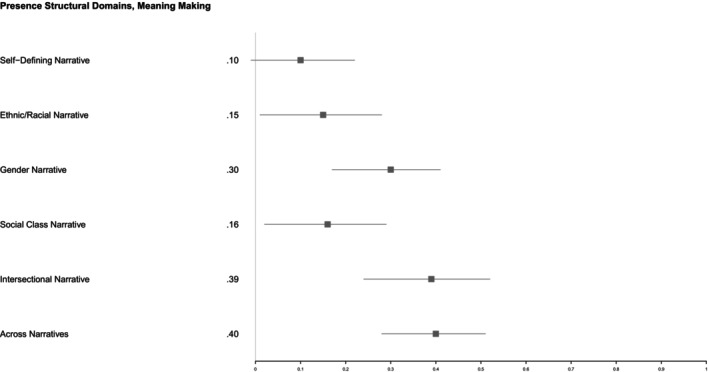
Partial correlations between meaning making and presence of structural domains controlling for narrative length. *p < 0.05.

There was also a positive correlation between these narrative features when participants' scores of *Meaning Making* were averaged across all narratives and examined in relation to the average number of structural domains mentioned across all narratives (Figure 5). It is worth noting that we also found a positive correlation between *Meaning Making* and *Connection Between Domains* in the intersectional narrative, while controlling for narrative length (*r* = 0.39, *p* = 0.00, 95% CI [0.23, 0.52]). There were no relations between *Affective Tone* and *Presence of Structural Domains* within or across narratives (Figure S1).

These relations demonstrate that *Meaning Making* is particularly important in narratives involving multiple structural domains and that this process may provide insight into the personal impact of identity intersections. For example, Isabelle, a Latine woman (objective social class = 0.19), evinced high *Meaning Making* in her intersectional narrative, sharing,

In high school, I was part of a program called Latina leaders. Being part of that organization was beneficial for me identifying strongly with being Mexican, being a woman, even my social class. We shared a lot of the same struggles being part of that social class. It felt good to be part of a group that I could share experiences with. Obviously, some of those experiences were negative, but still coming out and saying this is who we are, and we're stronger for that. Feeling empowered and strong, mentally, and physically. It was comforting. At times it feels like you're the only person who is going through this or you can't tell anybody because no one would understand. Those girls really do understand. I became who I really was after that. When I first entered the program, I didn't feel like I was good enough. Going through this program with those girls and having my identity of being Mexican, female, and part of a social class that I was, I feel like it embraced that part of me. These are things about you. All these experiences of those things make you who you are. When I came out of that program, I was a different person.

Through Isabelle's narrative, we see that interconnections among ethnicity/race, gender, and social class can function as sources of empowerment. *Meaning Making* of experiences involving multiple domains might make these domains more salient to the person and augment feelings of belonging. The connection garnered through shared experiences could be an integral part of the story for minority group members that empowers them to advocate for their community (Adler et al. 2022; Syed and McLean 2022). In fact, this participant noted, “Now that my identity was clear, I was able to help other people in my group.”

## 4 Discussion

To gain a comprehensive understanding of personality, it is critical to examine variations in lived experiences based on structural domains including ethnicity/race, gender, and social class (Galliher, McLean, and Syed 2017; Rogers, Moffitt, and Jones 2021). In this study, we integrated an intersectional framework and life narrative data to explore features of young adults' narratives about a domain‐general as well as ethnic/racial, gender, social class, and intersectional experience. Although this study was exploratory and descriptive in nature, helping to generate hypotheses for future research, our findings highlight that features of narratives vary based on experiences with structural domains and that structural domains have implications for a process of identity (i.e., *Meaning Making*). Below, we discuss these key takeaways as well as review avenues for future research and study limitations.

### 4.1 Variations in Narratives Based on Structural Domains

Through exploring young adult's life narratives around structural domains, we found that features manifested differently across narrative prompts. Each narrative prompt, except the self‐defining narrative, represented an experience with a structural domain. Thus, our findings provide preliminary evidence that young adult's experiences are aligned with and vary meaningfully on the basis of such domains. Notably, we did not find variations in narrative features based on self‐identified ethnicity/race, gender, and social class, except for *Affective Tone*, which was more positive for men participants. It is possible that men tell more positive stories based on structural privileges that enable fewer negative experiences with these domains. Still, our findings underscore that treating structural domains solely as categories of difference may be insufficient for assessing power dynamics (Azmitia et al. 2023). To understand how structural domains function in young adults' experiences, it is crucial for future work to continue examining variations in their emergence both within and across lived experiences.

Features may have differed across narratives due to systematic variability in how structural domains influence identity (see also McLean et al. 2016). For example, we found that the mention of ethnicity/race traversed narratives about multiple domains. In contrast, the mention of gender and social class were relatively more contained to narratives about those domains. This is consistent with past research indicating that experiences with gender and social class may be less salient, perhaps due to dominant narratives about gender equality and equal opportunity in the United States (McLean et al. 2016; Radmacher and Azmitia 2013). On the other hand, ethnicity/race may have been more salient for young adults in college, especially since data were collected in 2020, amid the COVID‐19 pandemic and societal events that raised awareness of ethnic/racial disparities (Rogers, Moffitt, and Jones 2021).

In addition to variations in the *Presence of Structural Domains*, we also found differences in young adults' connection to those domains in narratives. Specifically, *Connection to Structural Domain* was higher in ethnic/racial and gender narratives relative to social class narratives. It may be that young adults have greater difficulty forming connections with their social class. This is consistent with past research finding that class is often treated as an “invisible identity,” which may make connections with this domain more challenging to navigate (Radmacher and Azmitia 2013). Connection to social class may have different implications for identity and, perhaps, psychosocial functioning, relative to connections to one's ethnicity/race and gender. Indeed, we found that social class narratives were more negative relative to ethnic/racial and gender narratives, indicating that becoming aware of one's class might be more likely to involve difficult experiences. It is possible that negative experiences with lower connection (e.g., being dependent on others for resources) were more prevalent in this study because participants came from relatively lower social class backgrounds. While young adults might express pride in their ethnicity/race or gender as a means of resisting societal oppression, this strategy might not apply equally to the domain of social class. Based on how connection with social class manifested in this study, it may be that attributions of growth or change are more important for forming positive connections with this domain (Kraus, Piff, and Keltner 2009). It is also the case that our sample represented primarily ethnic/racial minority women, who are, perhaps, more attuned to challenges with social class, given interlocking structures of oppression (Collins 2019; Juan, Syed, and Azmitia 2016).

Although *Connection Between Structural Domains* was only assessed in one narrative, there were variations in how this feature emerged within intersectional narratives. Narrative data indicated that recognizing connections between structural domains may involve a greater awareness of interlocking structures of oppression. This is also supported by the positive correlation we found between *Connection Between Structural Domains* and *Meaning Making*. The extent to which young adults view structural domains to be connected may influence how they make sense of those domains. These meaning making processes may, in turn, impact the extent to which one recognizes and act against structural inequalities (i.e., form a sense of critical consciousness; Mathews et al. 2020). Additional research is needed to determine whether *Connection Between Structural Domains* in narratives might promote critical consciousness, particularly among young adult women of color. Such work would benefit from coding for oppression and resistance within narratives (Rogers and Syed 2021), as it is clear from this study that these features are prevalent in narratives about structural domains, even when not directly assessed.

We also found differences in the process‐oriented feature of *Meaning Making* across narratives. Specifically, we found that *Meaning Making* was lower in intersectional narratives relative to other domains and higher within self‐defining narratives. These findings align with past research indicating that young people may struggle to deeply consider connections between more than two identity domains *when asked directly* (Azmitia et al. 2023). Narrative prompts assessing domain‐general experiences or that capture interconnections among identities indirectly may be more useful for understanding how people process experiences involving interconnected identities and structural domains (Syed and McLean 2022). Building on these insights, our study emphasizes the need for researchers to refine narrative measures to better account for the role of prompt type and perhaps, reconsider examining relations with features aggregated across different prompts. The same narrative feature may function differently based on what the participant is asked to discuss. Although past research indicates that variations in narratives based on prompt type can exceed person‐level variability (McLean et al. 2017), few studies explicitly address this (but see Adler et al. 2015; McLean et al. 2020). Future research is needed that disentangles the role of prompt type and continues to develop prompts for assessing intersectional identity.

### 4.2 Interconnected Nature of Structural Domains

The findings of this study also expand past research examining intersecting identities by demonstrating how narratives about one structural domain carry implications for other domains and identity processes. For example, we found that even when not prompted, individuals use structural domains to guide their understanding of the self and interactions with the world. In turn, the content of their experiences with structural domains, particularly the extent to which multiple domains were incorporated, correlated positively with *Meaning Making* of life narratives. This finding builds upon and extends past work finding that the co‐occurrence of life domains can contribute to identity work (McLean et al. 2016) and shows how the interconnected nature of structural domains may contribute to processes of identity (Rogers, Niwa, et al. 2021). These findings are also consistent with past research indicating that unpacking the content of life narratives is critical for understanding identity development (Galliher, McLean, and Syed 2017; Syed and Azmitia 2010).

Ultimately, this study demonstrates that life narratives can enrich understanding of diversity in personality (Sabik 2016; Westberg and Syed 2024). Our results provide preliminary evidence that fusing life narrative data and an intersectional framework is a worthwhile endeavor, as it provides insight into how individuals actively navigate social structures and ways this might impact their identity. Through continuing to leverage narrative data, psychologists can perhaps gain a better understanding of what and how personality processes are informed by intersecting structural domains (Buchanan and Wiklund 2021; Moradi and Grzanka 2017).

## 5 Limitations and Future Directions

Although this study possesses several strengths, it is important to review limitations and provide recommendations for future work. Our sample was mostly composed of ethnically/racially diverse young adults from lower socioeconomic backgrounds, who are underrepresented in the psychological literature. Still, future work should examine how structural domains impact personality across the lifespan and among non‐college students. The current sample was also gathered at a Hispanic serving and Asian American and Pacific Islander serving university and over 40% of students at this university were eligible for a Pell grant (i.e., parental household income was < $50,000 per year). While this, in many ways, made the location ideal for our study, past research finds students from marginalized groups make sense of cultural experiences differently when they are the numeric minority (Syed and Azmitia 2010). Thus, our findings may be less generalizable to contexts with lower ethnic/racial and social class diversity.

Our narrative data represented a significant investment and was much larger relative to past studies exploring multiple identity domains using qualitative data. But researchers interested in more advanced quantitative analyses should recruit larger samples, perhaps through collaborative multi‐site efforts. Future work with larger samples could test how narratives are nested within prompts using multilevel modeling and further validate the narrative features examined in this study as well as others that were not explicitly assessed (e.g., oppression and resistance). The current study represents one step toward understanding better how structural domains are interconnected within young adults' experiences and may impact their identity development.

## 6 Conclusion

In this study, we integrated an intersectional framework and narrative identity approach to shed light on how narratives around structural domains including ethnicity/race, gender, and social class are interconnected. Using qualitative and quantitative analytic techniques, our findings reveal how structural domains are embedded in young adults' experiences and some of their implications for identity. Ultimately, this work strengthens our understanding of how personality is influenced by larger structures and advances ways researchers might seek to assess intersectionality within personality science.

## Author Contributions

**Dulce Wilkinson Westberg:** Conceptualization, Data Curation, Formal Analysis, Investigation, Methodology, Software, Writing – Original Draft Preparation, Writing – Review & Editing. **Moin Syed:** Supervision, Formal Analysis – Support, Writing – Review & Editing. **Aerika Brittian Loyd:** Supervision, Writing – Review & Editing. **William Dunlop:** Supervision, Conceptualization, Writing – Review & Editing.

## Conflicts of Interest

The authors declare no conflicts of interest.

## Supporting information


**Data S1.**.

## Data Availability

Researcher codes of qualitative data, analysis code, and study materials are available at https://osf.io/6zn4j/. Qualitative data are not made publicly available but are available to request from the corresponding author. This study's design and its analysis are exploratory and not preregistered.

## Endnotes

^1^
We intentionally lowercase the term white to emphasize that ethnicity/race are not neutral categories; rather, they are structural domains reflective of unequal hierarchies that have historically reinforced white supremacy.

^2^
This included two publications in *Personality and Social Psychology Bulletin* (Hall, Galinsky, and Phillips 2015; Martin and Slepian 2018), one publication in *Journal of Personality and Social Psychology* (Petsko, Rosette, and Bodenhausen 2022), and one publication in *Social Psychological and Personality Science* (Remedios, Reiff, and Hinzman 2020).

^3^
No participants identified only as Native/Indigenous or American Indian. Participants' specific pan‐ethnic identifications are available in the Table S1.

^4^
Participants were originally prompted for a moment of gender awareness, but this was changed to a salient moment with gender after the first week of data collection. This was done to capture an experience when gender was important or relevant to the participant, rather than the moment participants determined their gender identification. The full interview, including the original and final wording of the gender narrative, is located here.

^5^
The master coder approach is appropriate in cases when expert evaluation would benefit the analysis. In this case, the first author gathered most of the narrative data and coded all narratives for each feature; thus, she had the deepest understanding of the narrative data and could ensure high standards for qualitative analysis (Syed and Nelson 2015).

^6^
Given the inherent complexity of measuring social class, we opted for objective indicators of parental income and education, supported by past research showing their relevance among young adult college students. Our approach aligns with recent research emphasizing the need for a decision‐making process in measuring social class, tailored to the sample and research questions rather than relying on a single, definitive method (Antonoplis 2023).

^7^
This test and percentage were selected after the observed percentages were already known. The results were meant to be presented as descriptive only, but a reviewer insisted on an inferential test. We admit that 10% is arbitrary but seemed reasonable to us. Readers are welcome to apply their own threshold and draw their own conclusions.

^8^
Partial correlations controlling for narrative length remained the same or differed by 0.1 from the raw correlations.
