# Supplementary material for: Using Intersectionality to Understand How Structural Domains Are Embedded in Life Narratives
Source: J Pers. 2024 Nov 2;93(4):913–28. doi: 10.1111/jopy.12984 (PMC12224561; doi:10.1111/jopy.12984)
Supplement: Supplementary file 1 — Data S1.. [file JOPY-93-913-s001.docx]

**Supplemental Materials**

**Table S1**

*Open-Ended Ethnic/Racial Identification Responses*

|  | *N* |  | *N* |  | *N* |  | *N* |  | *N* |
| --- | --- | --- | --- | --- | --- | --- | --- | --- | --- |
| **Asian American** | 68 | **Latine** | 63 | **Multiracial** | 23 | **White** | 16 | **Black/African** | 6 |
| Asian | 18 | Chicana/o | 2 | Black, Filipino | 1 | Pakistani | 3 | African | 1 |
| Cambodian,  Chinese | 2 | Hispanic | 8 | German, Mexican | 1 | Lebanese | 2 | African, Black | 2 |
| Chinese,  Taiwanese | 1 | Hispanic,  Mexican | 1 | Iranian, Mexican | 1 | Middle Eastern | 1 | Black | 1 |
| Filipino | 9 | Latina/o | 7 | Italian, Mexican | 1 | Persian | 1 | Black, Nigerian | 1 |
| Singaporean | 1 | Mexican | 28 | Latinx, White | 1 | Egyptian | 3 |  |  |
| Vietnamese | 14 | Chicano,  Mexican | 2 | Mexican, Native | 1 | Finnish | 1 |  |  |
| Asian, Chinese | 1 | Guatamex | 1 | Mexican, White | 1 | Italian | 1 |  |  |
| Chinese, Filipino | 1 | Hispanic,  Latina/o | 6 | Hispanic, White | 2 | White | 4 |  |  |
| Filipino,  Vietnamese | 1 | Mexican, Latina | 1 | Black, Filipino, White | 1 |  |  |  |  |
| Japanese | 2 | Mexican,  Salvadorean | 2 | Black, Pacific Islander | 1 |  |  |  |  |
| Korean | 5 | Latinx | 2 | Cambodian, Chinese, Mexican | 1 |  |  |  |  |
| Chinese | 1 | Peruvian | 1 | Latina/o, White | 3 |  |  |  |  |
| Indian | 11 |  |  | South Asian, White | 1 |  |  |  |  |
| Taiwanese | 1 |  |  | Black, Hispanic | 1 |  |  |  |  |
|  |  |  |  | Black, White | 1 |  |  |  |  |
|  |  |  |  | Chinese, Mexican | 2 |  |  |  |  |
|  |  |  |  | Haitian, Indian | 2 |  |  |  |  |
|  |  |  |  | Mixed | 1 |  |  |  |  |

*Note.* This table represents the ethnic/racial identification that each participant provided in the open-text response box. One participant did not provide information about ethnicity/race. 10 Asian American, 2 Latine, and 1 Black participant did not report open-ended responses and were categorized based on the forced-choice assessment.

**Table S2**

*Narrative Prompts*

| **Self-Defining Memory Prompt** | My first question concerns the recall of a special kind of personal memory called a self-defining memory. To understand best what a self-defining memory is, imagine you have just met someone you like very much and are going for a walk together. Each of you is very committed to helping the other get to know the “Real You”. You are not trying to play a role or to strike a pose. While, inevitably, we say things that present a picture of ourselves that might not be completely accurate, imagine that you are making every effort to be honest. In the course of the conversation, you describe a memory that you feel conveys powerfully how you have come to be the person you currently are. It is precisely this memory, which you tell the other person and simultaneously repeat to yourself, that constitutes a self-defining memory.  I would now like you to please describe this memory. Please be as detailed as possible. What happened? Who was there? How did this event make you feel? Why is this event important to you? |
| --- | --- |
| **Ethnic-Racial Awareness Prompt** | Next, please describe a specific experience from your life when you became particularly aware of your ethnic-racial group. Please describe this event in as much detail as possible. When did this event occur? What were you thinking and feeling?  [Clarification: This is not necessarily the first time you became aware of your ethnicity/race but may be an experience from life when your ethnicity/race was particularly salient to you.]  Did this event affect what you think about or how you view your own ethnicity and race or ethnicity and race in general? **[5 minutes total for entire question]** |
| **Gender Salience Prompt** | Next please describe a salient memory that is relevant to your gender or gender identification. This may be a positive memory or a negative memory, it only matters that this event be an emotionally important experience for you. Please describe this experience in as much detail as possible. Who was there? What were you thinking and feeling? Why is this memory important to you? **[5 minutes total for entire question]**  [rephrase for participant based on their gender identification e.g., please describe a specific experience from your life when you became particularly aware that you were a {woman}, that you were {female}].  Did this event affect what you think about or how you view your own gender or gender in general? |
| **Social Class Awareness Prompt** | Next, can you please describe a specific experience from your life when you became particularly aware of your economic background or social class? Please describe this event in as much detail as possible. When did this event occur? What were you thinking and feeling?  Did this event affect what you think about or how you view your own economic background and social class or economic backgrounds and social class in general? **[5 minutes total for entire question]** |
| **Intersectional Awareness Prompt** | I have asked you questions pertaining to your life as a member of your ethnic/racial group, gender, and economic background. However, there are many experiences that are relevant across these social groups. I would like to take a moment to consider how you see yourself across these social groups.  Thinking back over your life, please identify a specific experience that describes who you are as a member of your ethnicity/race, gender, and economic background or social class. For example, a person who identifies as Black, female, and middle class may describe an experience that defines who she is across each of these domains. If you feel that your life contains no such event, then describe a particular episode in your life that comes closer than any other to describing how you think of yourself as a member of each of these groups. When did this event occur? What were you thinking and feeling? Do you feel that your identities were harmonious or opposing? Why is this event important to you? **[5 minutes total for entire question]** |

**Table S3**

*Narrative Features and Examples*

| **Feature** | **Description** | **Coding Scheme** | **Example** |
| --- | --- | --- | --- |
| **Meaning Making**  ICC = .73 | Degree to which one narrates self-understanding based on life experiences and uses this knowledge to guide behavior and/or thoughts about the self, others, or the world | 0 = No explanation of meaning of event  1 = Lesson learned from event  2 = Vague meaning. Some growth/change, but specifics are unclear  3 = Narrator gleaned specific insight from event that applies to broader areas of their life | 0 = “I am Mexican, female and middle class. I'll refer back to the quinceañera. Not lots of people are able to afford to have those types of parties and celebrations. We think that lots of girls who can’t, like those types of parties would feel kind of down and think less of themselves and their culture.”  *Intersectional Awareness; Mexican, Female, Middle Class*  3 = “When I played soccer, I was surrounded with a bunch of kids from different areas and ethnicities. When I personally was able to interact with kids from different ethnicities, we had discussions about our race, and what we will do on the daily being Hispanic or Black or Chinese or White. We all had different lives, examples, and experiences to talk about. However, being in a scenario where I was able to express myself, I was able to talk about being a Hispanic male, who didn't have a lot of money. Being in a scenario where I was able to talk about those things made me open my eyes to different options for myself. I was able to talk about not having a lot of money. I remember thinking this is a different outlet for myself. There's more jobs and opportunities, and justice. That was a scenario where it was okay to be a Mexican, Hispanic. That's a position that I will never forget because I was openly accepted, and not criticized or judged. I was offered new ideas.”  *Intersectional awareness; Mexican, Male, Working Class* |
| **Affective Tone**  ICC = .74 | Positive relative to negative emotional content of the narrative as a whole | 1 = Very negative  2 = Negative  3 = Neutral  4 = Positive  5 = Very positive | 1 = “One of the first experiences where I became aware of my background was on the playground. Me and my cousins interacting with new kids and introducing my cousin as my cousin. And people being surprised or kind of off put by that. It made me realize that we look different, and that I wasn't only Filipino. Growing up, I felt more culturally Filipino. I thought, that's all I really was. My family invested that in me and told me this was who to identify as, and this is where we're from. Even though my skin color was darker, I didn't really put two and two together that this was more of my identity than I identified it as. I think I was like, put off I was kind of sad, because I was like, why are you confused? For me, it was so apparent my whole life that I was Filipino because of what I ate how I spoke the language, how I spoke to my family, and that’s how I saw myself. It was really sad and hurtful being told that it was an impairment. It wasn't obvious that I was Filipino. I think it kind of made me feel less than or not as Filipino in a sense.”  *Ethnic/racial awareness; Filipino, Female, Middle Class*  5 = “Alright, probably the time I took a trip with my brother. We flew out to [redacted] to go to a music festival. I would say we just went. We didn't even book a hotel or anything. We literally slept in an airport or didn't sleep. We were just awake for like 48 hours, enjoying it. We got there and had the time of our lives. It was literally just us too. That made me feel really euphoric. It's kind of combining like sharing a hobby with my brother, who I'm really, really close with. It really made me feel alive. In a sense, I think it has things that are important to me. I have a twin brother, and he's kind of my life. It was probably one of my favorite artists. I think it was just a combination of happy memories and things that are important to me.”  *Self-defining memory; Mexican, Male, Upper Middle Class* |
| **Connection to Structural Domain**  ICC = .72 | Degree to which one narrates feeling connected relative to disconnected from the structural domain being discussed (i.e., ethnicity/race, gender, social class) | 1 = Disconnected  2 = Neither connected nor disconnected  3 = Connected | 1 = “When I was growing up, I grew up here in America and my mom and dad were both born in Mexico. I remember, we would go to Rosarito, or Tijuana sometimes, and we would just go and experience the life there. I remember being like, Oh my gosh, I'm missing out. I want to see Mexico. My parents, they're just not comfortable, bringing us there because of all the things that can happen. I learned later that my mom never wants us to go to Mexico where she grew up, because she's scared of what could happen because we're not from there. I remember being sad I was missing out on truly feeling Mexican. Growing up, Spanish is my first language. My mom would go to work and my grandma would take care of us. We didn't go to preschool because of circumstances. I would stay home speaking Spanish. Then as I got older, I just lost it. I felt like I was missing out. I realized that my mom does it for a reason. She wants what's best for us. It makes me sad. That if she wants what's best for us, we can't have that connection, the same way that she has to Mexico. It's heartbreaking to be Mexican, but not really feel it.”  *Ethnic-racial awareness; Mexican, Female, Middle Class*  3 = A vivid memory I have of being female was somewhat recent. I was shadowing my dad, who's an anesthesia tech, because I'm interested in the med field. I was watching a surgery, and I noticed as soon as I walked into the OR, it was just a bunch of guys, and like, two girls probably. The way the guys would interact with the girls disheartened me because they were really rude. The females were just trying to help them out, obviously, because it's a team. You're all supposed to be working as a team. And the guys are just being really rude and acting like, I know it all and you know nothing. That didn't sit well with me. It is something that drives me to continue my medical field track because it really bothers me how I feel like there's not enough women in STEM. And if there are women in STEM, they're not taken seriously. I remember in the moment, I was confused, because I had heard about women in STEM not having enough power and being not appreciated as much. And then I saw it firsthand. I was really shocked. I didn't think this was actually true. I came from an all-girls high school. So strong women is something that we really talked about and was really preached upon. Coming from that background, and then seeing that was heartbreaking. I believe it matters for me, because this is the future I want to go into and seeing how some of the women are being treated is absolutely disgusting. If anything, they did the same amount of school, they have the same level of education for some guy to be like, no, you're not doing this right. Even though they're on the same education level really angers me. It drives me to do better and study harder. Even though it disheartens me in a way. I think because I have my strong background on woman empowerment from my high school, it encourages me to encourage others for there to be more women in STEM because if anything I feel like it's overpowered by guys and I'm not a fan of that because women have equal to or more capabilities. It shows me that I need to empower other younger girls to do the same.”  *Gender salient memory; Filipino, Female, Upper Middle Class* |
| **Connection Between Structural Domains** ICC = .77 | Degree of connection relative to disconnection that one narrates between structural domains | 1 = Highly disconnected  2 = Disconnected  3 = Neither connected nor disconnected  4 = Connected  5 = Highly connected | 1 = “My cousin, he's male. He's also Indian. But in our community, at least, depression, anxiety, and burnout isn't really talked about. He was a pre-med college student trying to pursue a career in medicine. He fell into depression, he fell into stress, a lot of anxiety, burnout. He didn't have that support from his parents, his peers, and as a male, because we often talk about this, as a male, he felt as if he shouldn't be feeling this way. It was wrong for him to feel this way because of what society said. He'd say, screw what our parents say. Screw it. What our religion says, but like society in general says, us as men, we're not allowed to talk about things, not allowed to show weakness, we're not allowed to cry, we're not allowed to show any weakness. We show pain we’re considered weak. We’re considered inadequate to care for our families. I'll talk to him just let him know, Hey, just because we may have a problem, we may feel pain doesn't make us weak. That just makes us stronger. It makes us stronger on how we handle it, and you're handling it perfectly. You're reaching out to people you can trust, and you should continue reaching out to people you can trust. Nevertheless, he did not have that support group, and it ultimately led him to commit suicide. That's what made me think about my gender, my ethnic background, and I feel my social class as well, just all three coupled together. As a male, we're not allowed to talk about our feelings.  Not allowed to talk, we're not allowed to be in pain, we're not allowed to show that we can feel pain, we're not going to show ourselves as weak. I don't agree with that.” *Intersectional awareness; Indian, Male, Working Class*  5 = “The event that I can think of that kind of intertwined with all those three, like my race, economic class, and gender was folklorico dancing in elementary school. Everybody was taught this dance in our grade. I think it's from certain parts of Mexico that the dance originates from. It made me feel our community, we're getting involved with our race. It made me feel involved. It felt like all those three were being brought out like where we're from, you know, like we're not from a high class. We don't come from a high-class community, but we can still gather and, make these small events and work with what we have and perform a show. It also involves a little bit of our culture and stuff like that. That made me feel like we're getting involved. The females, they had to wear these big dresses, and the guys might be dressed up like a cowboy. It kind of set the tone like gender roles, but at the same time, it was nice to see the beautiful dresses that the females worn. How the men were chivalrous.” *Intersectional awareness; Mexican, Female, Working Class* |
| **Presence of Ethnicity/Race**  κ = .97 | Indicates whether participant mentioned ethnicity/race in their narrative | 0 = Absent, 1 = Present | -- |
| **Presence of Gender**  κ = .95 | Indicates whether participant mentioned gender in their narrative | 0 = Absent, 1 = Present | -- |
| **Presence of Social Class**  κ = .96 | Indicates whether participant mentioned social class in their narrative | 0 = Absent, 1 = Present | -- |

*Note*. Narratives were edited for brevity. ICC and κ represent the average reliability across the narrative prompts where the feature was quantified. *Meaning Making* was quantified in all narratives. *Connection to Structural Domain* was quantified in the ethnic awareness, gender salient, and social class awareness narratives. *Connection Between Structural Domains* was quantified in the intersectional awareness narrative. Examples are not provided for features including the presence of ethnicity/race, gender, and social class, as these were quantified based on whether these structural domains were mentioned by the participant

**Table S4**

*Regression Models Examining Differences in Average Presence of Ethnicity/Race, Gender, and Social Class Based on Participant Ethnicity/Race, Gender, and Social Class*

|  | Model 1 | | Model 2 | |
| --- | --- | --- | --- | --- |
|  | b (SE) | β | b (SE) | β |
| Presence of Ethnicity/Race |  |  |  |  |
| Intercept | .50 (.02)*** | - | .51 (.02)*** | - |
| Ethnicity/Race | -.03 (.02) | -.10 | -.04 (.03) | -.12 |
| *Covariates* |  |  |  |  |
| Gender |  |  | -.00 (.02) | -.02 |
| Social Class |  |  | -.00 (.01) | -.03 |
| Presence of Ethnicity/Race |  |  |  |  |
| Intercept | .49 (.02)*** | - | .51 (.02)*** | - |
| Gender | .00 (.02) | -.02 | -.00 (.02) | -.02 |
| *Covariates* |  |  |  |  |
| Ethnicity/Race |  |  | -.03 (.03) | -.12 |
| Social Class |  |  | -.00 (.02) | -.03 |
| Presence of Ethnicity/Race |  |  |  |  |
| Intercept | .48 (.01)*** | - | .51 (.02)*** | - |
| Social Class | -.01 (.02) | -.10 | -.00 (.02) | -.03 |
| *Covariates* |  |  |  |  |
| Ethnicity/Race |  |  | -.04 (.03) | -.12 |
| Gender |  |  | -.00 (.02) | -.02 |
| Presence of Gender |  |  |  |  |
| Intercept | .40 (.01)*** | - | .39 (.02)*** | - |
| Ethnicity/Race | -.04 (.02)* | -.18 | -.03 (.02) | -.13 |
| *Covariates* |  |  |  |  |
| Gender |  |  | -.00 (.02) | -.04 |
| Social Class |  |  | -.00 (.01) | -.02 |
| Presence of Gender |  |  |  |  |
| Intercept | .38 (.01)*** | - | .39 (.02)*** | - |
| Gender | -.00 (.02) | -.03 | -.01 (.02) | -.05 |
| *Covariates* |  |  |  |  |
| Ethnicity/Race |  |  | -.01 (.02) | -.06 |
| Social Class |  |  | -.00 (.01) | -.04 |
| Presence of Gender |  |  |  |  |
| Intercept | .38 (.01)*** | - | .40 (.02)*** | - |
| Social Class | -.00 (.01) | -.06 | -.00 (.01) | -.02 |
| *Covariates* |  |  |  |  |
| Ethnicity/Race |  |  | -.03 (.02) | -.13 |
| Gender |  |  | -.00 (.02) | -.04 |
| Presence of Social Class |  |  |  |  |
| Intercept | .45 (.02)*** | - | .44 (.02)*** | - |
| Ethnicity/Race | -.06 (.02)** | -.24 | -.04 (.02) | -.16 |
| *Covariates* |  |  |  |  |
| Gender |  |  | -.00 (.02) | -.05 |
| Social Class |  |  | -.02 (.02) | -.16 |
| Presence of Social Class |  |  |  |  |
| Intercept | .42 (.02)*** | - | .44 (.02)*** | - |
| Gender | .00 (.02) | .01 | -.00 (.02) | -.00 |
| *Covariates* |  |  |  |  |
| Ethnicity/Race |  |  | -.04 (.02) | -.16 |
| Social Class |  |  | -.02 (.02) | -.15 |
| Presence of Social Class |  |  |  |  |
| Intercept | .42 (.01)*** | - | .44 (.02)*** | - |
| Social Class | -.03 (.01)** | -.23 | -.02 (.01) | -.15 |
| *Covariates* |  |  |  |  |
| Ethnicity/Race |  |  | -.04 (.02) | -.16 |
| Gender |  |  | -.00 (.02) | -.05 |
| *Note. N* = 145, (75 Asian American, 70 Latinx; 63 male, 81 female, 1 non-binary participant excluded from gender analyses); Ethnicity/Race coded as 0 = Latinx, 1 = Asian American; Gender coded as 0 = male, 1 = female; Social class = aggregated z scores of parental income and parental education.  ^†^*p* = .10, ***p* = .01, ****p* = .001 | | | | |

**Table S5**

*Regression Models Examining Differences in Average Connection to Structural Domain Based on Participant Ethnicity/Race, Gender, and Social Class*

|  | Model 1 | | Model 2 | |
| --- | --- | --- | --- | --- |
|  | b (SE) | β | b (SE) | β |
| Connection to Domain |  |  |  |  |
| Intercept | 2.28 (.06)*** | - | 2.35 (.08)*** | - |
| Ethnicity/Race | .06 (.08) | .06 | .00 (.09) | .00 |
| *Covariates* |  |  |  |  |
| Gender |  |  | -.09 (.08) | -.10 |
| Social Class |  |  | .06 (.05) | .10 |
| Connection to Domain |  |  |  |  |
| Intercept | 2.35 (.06)*** | - | 2.35 (.08)*** | - |
| Gender | -.07 (.08) | -.07 | -.09 (.08) | -.10 |
| *Covariates* |  |  |  |  |
| Ethnicity/Race |  |  | .00 (.09) | .00 |
| Social Class |  |  | .06 (.05) | .10 |
| Connection to Domain |  |  |  |  |
| Intercept | 2.30 (.04)*** | - | 2.35 (.08)*** | - |
| Social Class | .06 (.05) | .11 | .06 (.05) | .10 |
| *Covariates* |  |  |  |  |
| Ethnicity/Race |  |  | .00 (.09) | .00 |
| Gender |  |  | -.09 (.08) | -.10 |
| *Note. N* = 145, (75 Asian American, 70 Latinx; 63 male, 81 female, 1 non-binary participant excluded from gender analyses); Ethnicity/Race coded as 0 = Latinx, 1 = Asian American; Gender coded as 0 = male, 1 = female; Social class = aggregated z scores of parental income and parental education.  ^†^*p* = .10, ***p* = .01, ****p* = .001 | | | | |

**Table S6**

*Regression Models Examining Differences in Average Connection Between Structural Domains in Intersectional Awareness Narratives Based on Participant Ethnicity/Race, Gender, and Social Class*

|  | Model 1 |  | Model 2 |  |
| --- | --- | --- | --- | --- |
|  | b (SE) | β | b (SE) | β |
| Connection Between Domains |  |  |  |  |
| Intercept | 3.57 (.15)*** | - | 3.44 (.22)*** | - |
| Ethnicity/Race | -.30 (.21) | -.12 | .20 (.24) | -.08 |
| *Covariates* |  |  |  |  |
| Gender |  |  | .03 (.22) | .01 |
| Social Class |  |  | .00 (.15) | -.00 |
| Connection Between Domains |  |  |  |  |
| Intercept | 3.34 (.16)*** | - | 3.44 (.22)*** | - |
| Gender | .09 (.21) | .04 | .03 (.22) | -.01 |
| *Covariates* |  |  |  |  |
| Ethnicity/Race |  |  | -.20 (.24) | -.08 |
| Social Class |  |  | .00 (.15) | .00 |
| Connection Between Domains |  |  |  |  |
| Intercept | 3.36 (.10)*** | - | 3.44 (.22)*** | - |
| Social Class | -.03 (.13) | -.02 | -.00 (.15) | -.02 |
| *Covariates* |  |  |  |  |
| Ethnicity/Race |  |  | -.20 (.24) | -.08 |
| Gender |  |  | .03 (.22) | .01 |
| *Note. N* = 145, (75 Asian American, 70 Latinx; 63 male, 81 female, 1 non-binary participant excluded from gender analyses); Ethnicity/Race coded as 0 = Latinx, 1 = Asian American; Gender coded as 0 = male, 1 = female; Social class = aggregated z scores of parental income and parental education.  ***p* = .01, ****p* = .001 | | | | |

**Table S7**

Regression Models Examining Differences in Average Meaning Making Based on Participant Ethnicity/Race, Gender, and Social Class

|  | Model 1 | | Model 2 | |
| --- | --- | --- | --- | --- |
|  | b (SE) | β | b (SE) | β |
| Meaning Making |  |  |  |  |
| Intercept | 1.96 (.07)*** | - | 1.93 (.11)*** | - |
| Ethnicity/Race | -.08 (.10) | -.06 | -.05 (.12) | -.04 |
| *Covariates* |  |  |  |  |
| Gender |  |  | .03 (.11) | .02 |
| Social Class |  |  | -.03 (.07) | -.04 |
| Meaning Making |  |  |  |  |
| Intercept | 1.89 (.08)*** | - | 1.93 (.11)*** | - |
| Gender | .03 (.10) | .02 | .03 (.11) | .02 |
| *Covariates* |  |  |  |  |
| Ethnicity/Race |  |  | -.05 (.12) | -.04 |
| Social Class |  |  | -.04 (.07) | -.04 |
| Meaning Making |  |  |  |  |
| Intercept | 1.92 (.05)*** | - | 1.93 (.11)*** | - |
| Social Class | -.04 (.06) | -.06 | -.04 (.08) | -.04 |
| *Covariates* |  |  |  |  |
| Ethnicity/Race |  |  | -.05 (.12) | -.04 |
| Gender |  |  | .03 (.11) | .03 |
| *Note. N* = 131, (68 Asian American, 63 Latinx; 58 male, 71 female, 1 non-binary participant excluded from gender analyses); Ethnicity/Race coded as 0 = Latinx, 1 = Asian American; Gender coded as 0 = male, 1 = female; Social class = aggregated z scores of parental income and parental education.  ^†^*p* = .10, ***p* = .01, ****p* = .001 | | | | |

**Table S8**

Regression Models Examining Differences in Average Meaning Making Based on Participant Ethnicity/Race, Gender, and Social Class

|  | Model 1 | | Model 2 | |
| --- | --- | --- | --- | --- |
|  | b (SE) | β | b (SE) | β |
| Affective Tone |  |  |  |  |
| Intercept | 2.92 (.07)*** | - | 3.08 (.10)*** | - |
| Ethnicity/Race | .04 (.09) | .04 | -.03 (.11) | -.02 |
| *Covariates* |  |  |  |  |
| Gender |  |  | -.30 (.10)** | -.23 |
| Social Class |  |  | .08 (.07) | .12 |
| Affective Tone |  |  |  |  |
| Intercept | 3.06 (.07)*** | - | 3.08 (.10)*** | - |
| Gender | -.24 (.09)* | -.22 | -.26 (.10)** | -.23 |
| *Covariates* |  |  |  |  |
| Ethnicity/Race |  |  | -.03 (.11) | -.02 |
| Social Class |  |  | .08 (.07) | .12 |
| Affective Tone |  |  |  |  |
| Intercept | 2.92 (.05)*** | - | 3.08 (.10)*** | - |
| Social Class | .09 (.06)* | .13 | .08 (.07) | .12 |
| *Covariates* |  |  |  |  |
| Ethnicity/Race |  |  | -.03 (.11) | -.02 |
| Gender |  |  | -.26 (.10)** | -.23 |
| *Note. N* = 131, (68 Asian American, 63 Latinx; 58 male, 71 female, 1 non-binary participant excluded from gender analyses); Ethnicity/Race coded as 0 = Latinx, 1 = Asian American; Gender coded as 0 = male, 1 = female; Social class = aggregated z scores of parental income and parental education.  ^†^*p* = .10, ***p* = .01, ****p* = .001 | | | | |

**Figure S1**

*
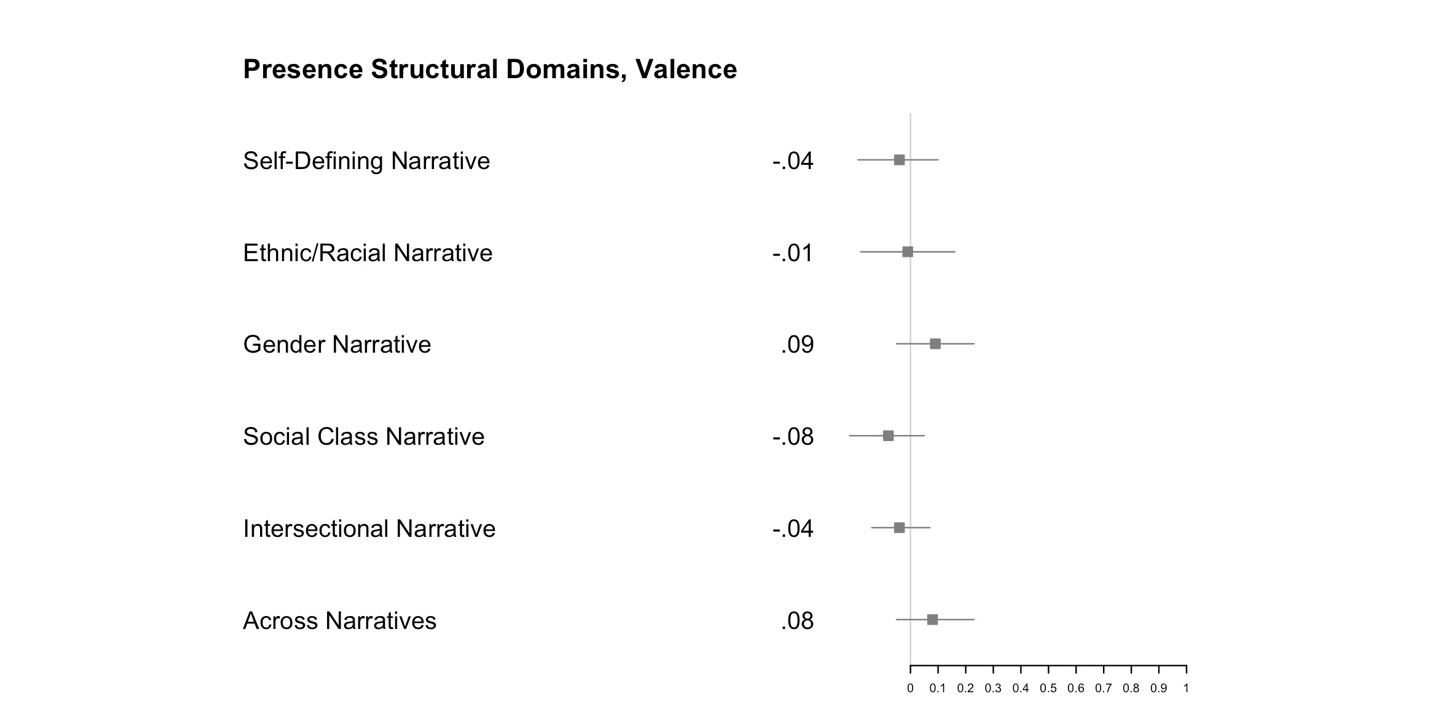
Partial correlations between Presence of Structural Domains and Affective Tone Controlling for Narrative Length*
